# Ultrasound-guided fine-needle cytology for cervical lymph nodes: a tertiary centre’s experience with the Sydney system

**DOI:** 10.1080/07853890.2025.2575301

**Published:** 2025-10-22

**Authors:** Xiaoyi Yan, Yanping Ding, Yang Gui, Li Tan, Jing Zhang, Qing Zhang, Xueqi Chen, Hua Liang, Bo Kong, Zhilan Meng, Xiaoyan Chang, Ke Lv

**Affiliations:** a Department of Ultrasound, Peking Union Medical College Hospital, Peking Union Medical College, Chinese Academy of Medical Sciences, Beijing, China; b Department of Ultrasound, Aerospace Center Hospital, Beijing, China; c Department of Pathology, Peking Union Medical College Hospital, Peking Union Medical College, Chinese Academy of Medical Sciences, Beijing, China

**Keywords:** Ultrasound-guided fine-needle aspiration cytology, ultrasound-guided fine-needle non-aspiration cytology, cervical lymphadenopathies

## Abstract

**Purpose:**

To evaluate the diagnostic value of ultrasound-guided fine-needle aspiration cytology (US-FNAC) and ultrasound-guided fine-needle non-aspiration cytology (US-FNNAC) on cervical lymphadenopathies, in which the authors specifically analysed the influence of lymph node size.

**Materials and Methods:**

A total of 500 lymphadenopathies were retrospectively enrolled from January 2019 to July 2023. The lymph nodes were divided into four size groups: ≤5.0 mm, from 5.1 to 10.0 mm, from 10.1 to 15.0 mm and >15.0 mm. The cytohistologic diagnosis was evaluated based on the Sydney System: I. inadequate/nondiagnostic; II. benign; III. atypical cells with uncertain significance/atypical lymphoid cells with undetermined significance; IV. Suspicious and V. malignant. The diagnostic yield of US-FNAC and US-FNNAC were assessed based on sensitivity (SEN), specificity (SPE), positive predictive value (PPV), negative predictive value (NPV) and accuracy calculations.

**Results:**

The overall SEN, SPE, PPV, NPV and accuracy of ultrasound-guided fine-needle cytology were 88.7%, 89.7%, 96.2%, 72.9%, 88.9%, respectively. The diagnostic accuracy and SEN of US-FNAC were superior to that of US-FNNAC in the overall cases (95.1% vs 83.9%, *p* < 0.001;95.6% vs 83.4%, *p* < 0.001) and in lymph nodes that measured from 5.1 to 10.0 mm(94.5% vs 85.1%, *p* = 0.022; 95.8% vs 84.4%, *p* = 0.021) as well as that from 10.1 to 15.0 mm (98.6% vs 86.0%, *p* = 0.011; 98.2% vs 83.3%, *p* = 0.011), while there was no significant difference between US-FNAC and US-FNNAC in the diagnostic yield among the other two subgroups.

**Conclusions:**

The current findings supported the preferential use of US-FNAC over US-FNNAC in routine clinical practice for lymph node evaluation, particularly for nodes measuring 5.1–15.0 mm. For lymphadenopathies ≤5.0 mm, additional tests were required to enhance the diagnostic performance of US-FNC, with US-FNAC often being necessary. Thus, we recommended using US-FNAC to obtain cytological specimens for definitive diagnosis of cervical lymphadenopathies that ≤15.0 mm.

## 1. Introduction

Differential diagnosis of benign and malignant lymphadenopathies in the neck through imaging techniques can be extremely challenging due to the frequently overlapping characteristics observed in lymphoma, lymphoproliferative disorders, metastatic cancer and inflammatory diseases. Misdiagnosis or missed diagnosis of cervical lymphadenopathies can severely impact patient outcomes, leading to unnecessary surgical interventions, delayed treatment of malignancies, or inappropriate follow-up strategies. Given that the selection of treatment strategy and prognosis rely on accurate characterization of the lesion, pathological confirmation is imperative for initial diagnosis and prior to non-surgical treatments [1,2]. Excisional biopsy is considered as a standard diagnostic procedure for certain lymphadenopathies, such as lymphoma [3]. However, it is time-consuming and may lead to relatively large scar [4]. Ultrasound-guided core needle biopsy (US-CNB) has been increasingly used for the high diagnostic accuracy [4]. Whereas the current method of performing US-CNB involves an automated biopsy device that has a fixed ejection distance, which makes it difficult to obtain tissue samples from small lymph nodes or those located near large cervical vessels [5].

Ultrasound-guided fine-needle cytology (US-FNC) plays an essential role in the diagnosis and clinical decisions of cervical lymphadenopathies for its simple, rapidity, minimum invasiveness as well as cost-effectiveness, especially for small lymph nodes [6]. The provided materials for ancillary testing such as flow cytometry (FC), immunocytochemistry (ICC) has further improved its diagnostic efficiency [7]. There are two commonly employed techniques for fine-needle cytology, namely fine-needle aspiration cytology (FNAC) and fine-needle non-aspiration cytology (FNNAC). FNAC was being used as the first diagnostic tool in the assessment of cytology. The fundamental principle involves the extraction of cellular material from target masses, often using relatively high suction pressures [8,9], which may result in blood contamination. FNNAC is an alternative technique that utilizes capillarity for sample collection developed in the 1980s, which is also known as fine-needle capillary sampling [10]. It eliminates the need for aspiration by a syringe and is thought to reduce tissue trauma and blood contamination [11]. However, FNNAC may yield fewer cellular samples, raising concerns about its diagnostic adequacy.

While FNAC and FNNAC have been extensively studied in the context of thyroid nodules, their comparative efficacy in cervical lymphadenopathies remains poorly understood [12–15]. Thyroid nodules and cervical lymph nodes differ significantly in aetiology, blood supply and diagnostic requirements. Thyroid nodules are typically homogeneous in origin, whereas cervical lymphadenopathies may arise from diverse causes, including infections, autoimmune diseases and malignancies [14,16]. Moreover, lymph node cytology often requires more extensive sampling for ancillary tests such as ICC and FC, making cellular yield and sample quality critical factors. Thus, whether the existing research results about FNAC and FNNAC on thyroid nodules are applicable to lymph nodes is unknown. There were limited data on lymph nodes related to the comparison of FNAC and FNNAC. Xia et al. reported that the two techniques may yield specimens with similar quality in the evaluation of lymph node metastasis of thyroid cancer [17]. An animal study found that FNAC was superior to FNNAC for the sampling of canine and feline lymph nodes as it generated a higher number of diagnostic samples with greater cellularity [18]. However, the number of cases in the above studies was relatively small and the types of lymphadenopathies were limited. In addition, lacking a universally accepted terminology for reporting lymph node cytology results also obstacle the application.

The Sydney System for lymph node cytopathology categorization and reporting was proposed by an international expert panel at the 20th International Congress of Cytology in 2019 [19]. Developed based on well-documented international cytopathology studies and the collective expertise of contributors worldwide, this system standardizes the classification of cytology results into well-defined diagnostic tiers. By improving communication between pathologists and clinicians, the Sydney System facilitates more accurate treatment planning and enhances diagnostic reproducibility. Initially designed for FNAC, subsequent studies have demonstrated its high diagnostic value when applied to FNNAC as well [5,6].

Therefore, this study aimed to compare the diagnostic value of ultrasound guided FNAC (US-FNAC) and ultrasound-guided FNNAC (US-FNNAC) in cervical lymphadenopathies, particularly when stratified by lymph node size. Additionally, we aimed to assess the applicability of the Sydney System for standardized classification and reporting in this context.

## 2. Materials and methods

### 2.1. Patients and study design

This study was conducted adhered to the principles of the Helsinki Declaration and approved by the Ethics Committee of Peking Union Medical College Hospital (I-22PJ1066). As this was a retrospective study, informed consent was waived by the Ethics Committee of Peking Union Medical College Hospital. Patients who underwent US-FNC between January 2019 and July 2023 were consecutively enrolled and retrospectively analysed at Peking Union Medical College Hospital. The lymph nodes were categorized into four size groups based on the smallest lymph node dimension (perpendicular to the longitudinal axis) observed on ultrasound: ≤5.0 mm, from 5.1 to 10.0 mm, from 10.1 to 15.0 mm, and > 15.0 mm. All relevant information, including patients age, sex, ultrasound imaging of lesions before biopsy, and final diagnosis were recorded. The exclusion criteria include [1]: the diagnosis could not be confirmed with FNC, CNB, surgery, or clinical follow-up [2]; patients without complete clinical information [3]; patients with repeated US-FNC in our hospital.

### 2.2. US-FNAC and US-FNNAC procedures

**Figure 1.**
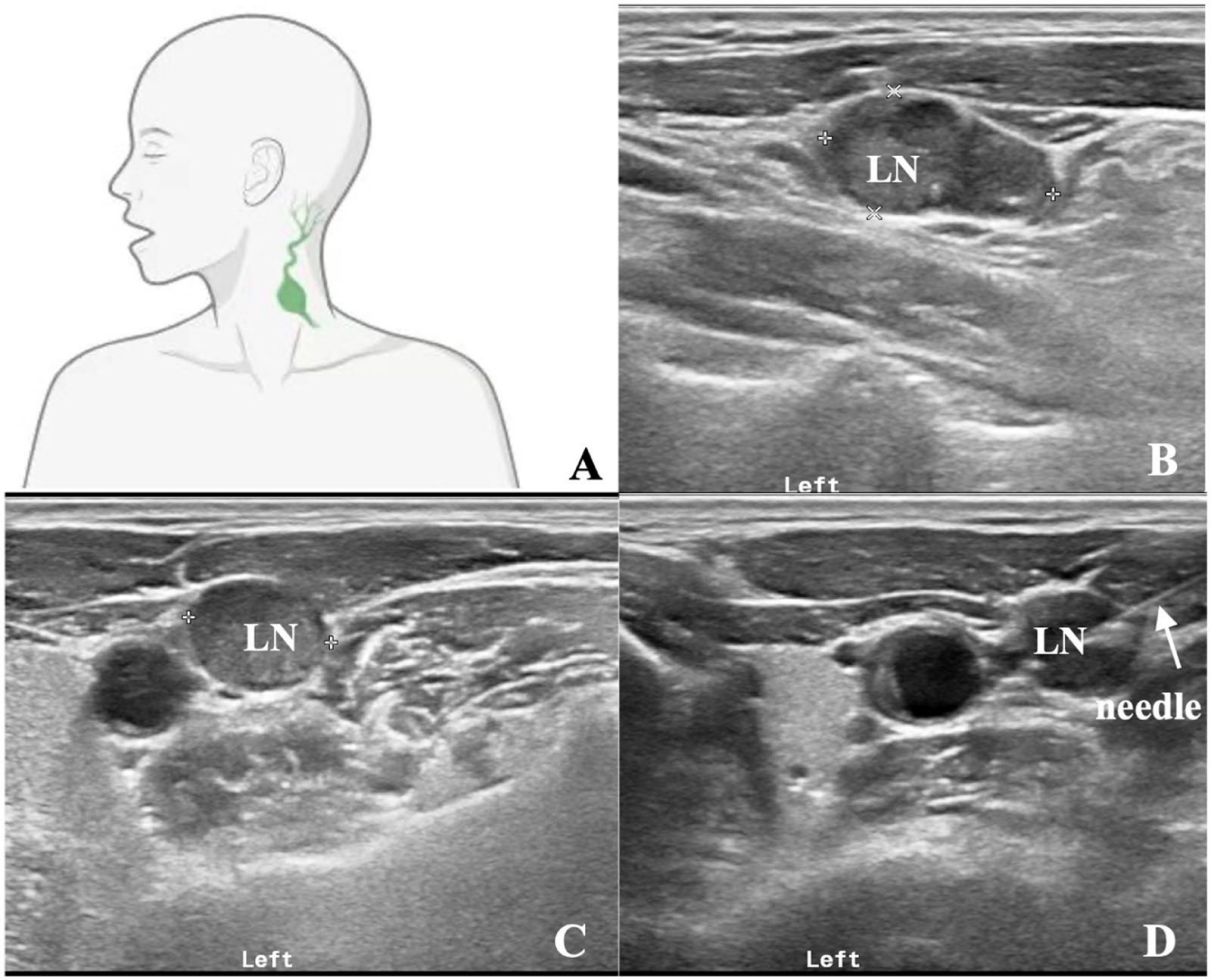
US-FNAC of an abnormal lymph node (LN) with microcalcification located near the carotid artery and jugular vein in the left side of the neck (**A** and **B**). Supine position was preferred, and the probe was in transverse scan (**C**). FNAC was successfully performed under the real-time guidance of ultrasound (**D**).

Prior to the biopsy procedures, all lesions were thoroughly reviewed by interventional (ultrasound) radio­logists (with minimum 10 years experience of ultrasound interventional experience). The size (based on the smallest lymph node dimension), location, microcalcification, the proportion of cystic areas (I. none; II. ≤50%; III. >50%) of the lymphadenopathy were evaluated. For patients with multiple lymphadenopathies, the most suitable one was selected for biopsy. Based on the size and location of the lymph nodes, the radiologists conducted a comprehensive evaluation to determine the suitability of the biopsy and its optimal approach (US-CNB, US-FNAC or US-FNNAC). As for US-FNAC, a 21-gauge needle connected to a 10-ml disposable plastic syringe was employed. During aspiration, the needle was inserted into the target lymph node and moved back and forth within the lymph node. Subsequently, suction was released, and the needle was carefully withdrawn; collected material was expelled onto glass slides and smears were meticulously prepared. US-FNNAC was performed as follows: the hub of a 23-gauge needle was held in a pencil-grip fashion, and the needle was gently inserted into the nodule and then moved in and out over 5 to 15 s. In cases where the differential diagnosis included large cell non-Hodgkin lymphoma, Hodgkin lymphoma, or metastases, remaining materials in the needle were preserved in 5 mL of 10% neutral buffered formalin for subsequent analysis such as microbiological examinations, FC or ICC, as deemed necessary. Figure 1 showed an example of the position and percutaneous approach in a patient with abnormal lymph node located near the carotid artery and jugular vein in the left side of the neck.

### 2.3. Cytologic diagnosis

The initial diagnoses underwent a thorough review by senior pathologists blinded to the sampling technique, and each case was evaluated based on the Sydney System: I. inadequate/nondiagnostic; II. benign; III. atypical cells with uncertain significance/atypical lymphoid cells with undetermined significance; IV. suspicious; V. malignant. Any inconsistencies in the categorization were addressed by reaching a consensus among at least two pathologists. Considering the heightened probability of malignancy associated with atypical cases, we have classified this group as positive diagnoses. Final diagnoses were determined based on [1]: histology from CNB or surgery [2]; diagnoses via FNC (including ancillary testing such as FC, ICC) [3]; for patients with negative or inadequate sampling, follow-ups were made every 6 months mainly through ultrasound, CT or PET/CT, the final diagnosis was established based on a minimum follow-up period of 18 months.

### 2.4. Statistical analysis

Continuous data were presented as mean ± standard deviation (SD) or median (interquartile range, IQR), while categorical variables were shown as frequencies and percentages. Diagnostic performance was assessed in terms of accuracy, sensitivity (SEN), specificity (SPE), positive predictive value (PPV), and negative predictive value (NPV), with 95% confidence intervals (95%CI) provided. Differences between the two techniques among the four size subgroups were analysed using appropriate statistical tests such as Student *t*-test, chi-square test, or Fisher exact test. Statistical significance was considered at a threshold of *p* < 0.05. MedCalc and SPSS software version 26.0 were used for statistical analysis.

## 3. Results

**Figure 2.**
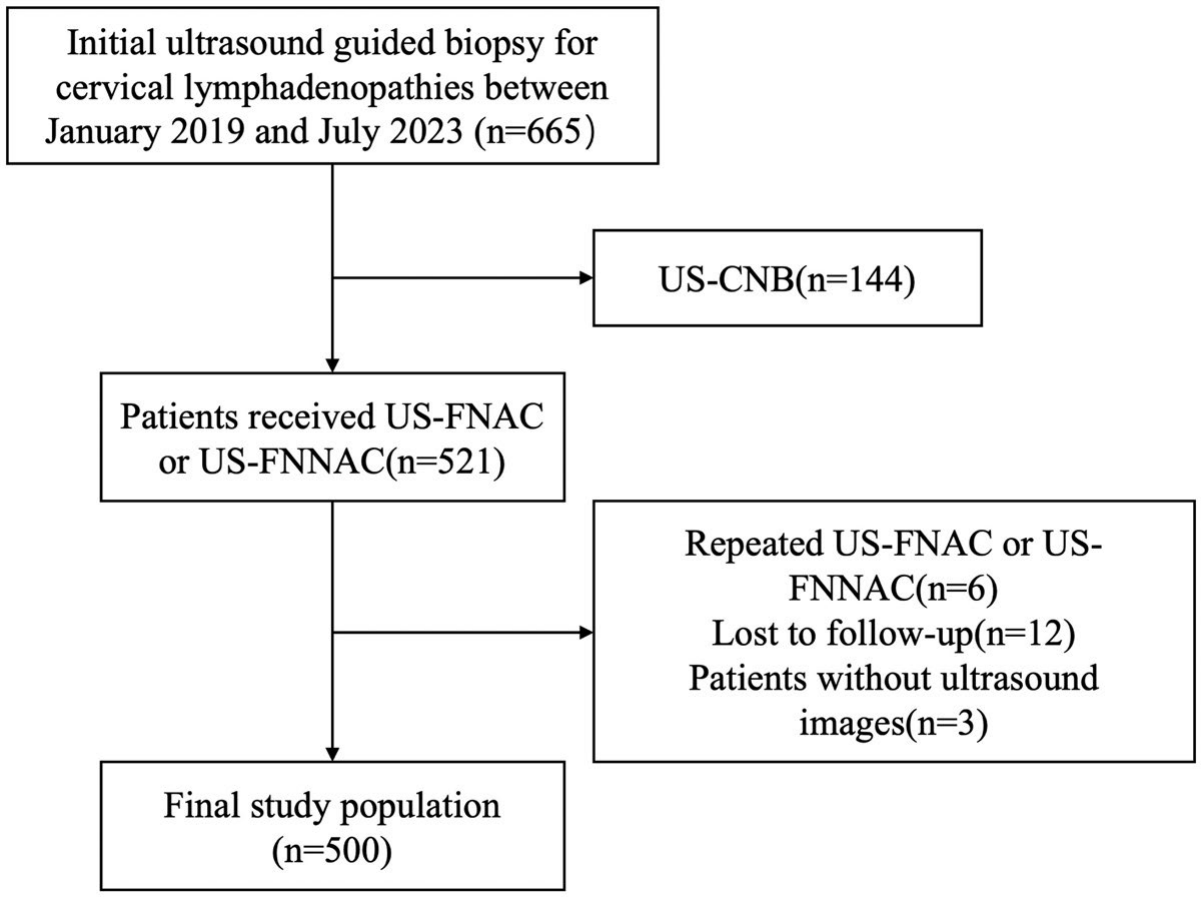
Flow chart of selecting the enrolled patients.

A total of 500 lymphadenopathies in 500 patients were finally enrolled in this study, in which 225 underwent US-FNAC and 275 underwent US-FNNAC. The patient selection flowchart was shown in Figure 2. Of the 500 lymph nodes (the smallest lymph node dimension: range, 3.0–53.0 mm; mean ± SD: 9.5 ± 0.5 mm), there were 104 lymph nodes ≤ 5.0 mm, 223 lymph nodes that measured from 5.1 to 10.0 mm, 129 lymph nodes that measured from 10.1 to 15.0 mm, and 44 lymph nodes >15.0 mm. The cytopathologic results from the different size groups for US-FNAC and US-FNNAC sampling were provided in Table 1.

**Table 1.** Cytologic results of fine-needle aspiration cytology and fine-needle non-aspiration cytology.

Cytologic Results	≤5.0 mm (*N* = 104)		5.1–10.0 mm (*N* = 223)		10.1–15.0 mm (*N* = 129)		>15.0 mm (*N* = 44)		Total
	FNAC	FNNAC	FNAC	FNNAC	FNAC	FNNAC	FNAC	FNNAC	
Nondiagnostic	0	1	0	0	2	0	0	0	3
Benign	10	35	38	36	15	17	3	2	156
Atypical	0	3	3	2	3	0	1	0	12
Suspicious	3	17	11	22	3	4	1	2	63
Malignant	9	26	57	54	49	36	17	18	266
Total	22	82	109	114	72	57	22	22	500

*FNAC*: fine-needle aspiration cytology; *FNNAC*: fine-needle non-aspiration cytology.

There were 128 benign lesions and 372 malignant lymphadenopathies. The distribution of cytopathological findings among the enrolled cases was as follows: 3 cases (0.6%) were classified as category I, 156 (31.2%) as category II, 12 (2.4%) as category III, 63 (12.6%) as category IV, and 266(53.2%) as category V. The diagnostic performance of the Sydney System for classification and reporting lymph node cytopathology demonstrated an overall SEN of 88.7%, SPE of 89.7%, PPV of 96.2%, NPV of 72.9%, and accuracy of 88.9% in distinguishing benign, atypical, suspicious, or malignant cytopathology. When atypical cases were excluded, the diagnostic performance of the Sydney System improved slightly, with a SEN of 88.5%, SPE of 93.3%, PPV of 97.6%, NPV of 72.7%, and accuracy of 89.7%. In comparison, FNNAC showed a SEN of 83.4%, SPE of 85.7%, PPV of 95.1%, NPV of 60.7%, and accuracy of 83.9%, while FNAC exhibited superior performance with a SEN of 95.6%, SPE of 93.7%, PPV of 97.5%, NPV of 89.4%, and accuracy of 95.1%. Statistical analysis revealed significant differences in SEN and accuracy between the two techniques (both *p* < 0.001), while no significant difference was observed in SPE (*p* = 0.143).

In our study, the benign lesions included granulomatous inflammation, reactive inflammation, Castleman disease, tuberculosis and Warthin tumour. The malignancies included metastases (thyroid cancer, lung cancer, breast cancer and ovarian cancer) and lymphoma. In the malignant lesions, 67 cases initially presented with a cytological diagnosis of atypical cells, suspicious, or malignant; however, the origin of the lesion or its pathological subtype could not be clearly determined. Following ICC examinations, definitive diagnoses were achieved in 64 cases, resulting in an upgraded diagnosis.

**Figure 3.**
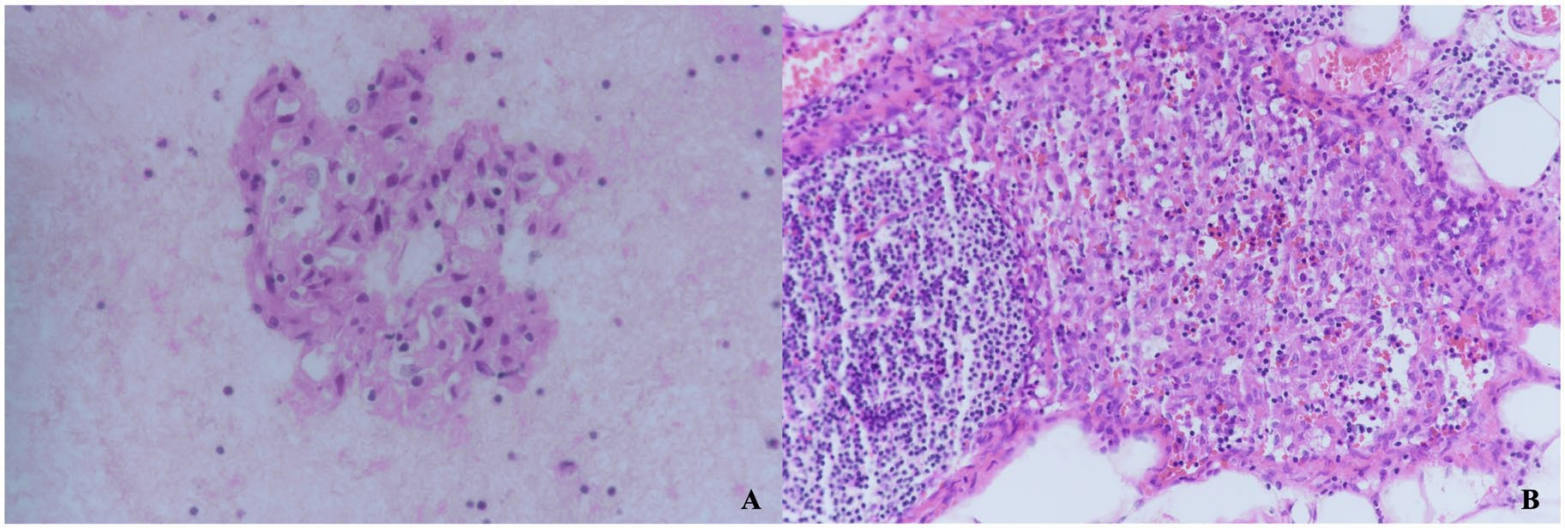
Cytological and histopathological findings in a 72-year-old female with suspected lymph node abnormalities. (A) FNAC revealed features suggestive of suspicious malignancy (40× magnification); (B) Surgical resection and immunohistochemical analysis confirmed granulomatous lesions (20× magnification).

A total of 14 patients were misdiagnosed by cytologic results, of which 5 cases using FNAC, and 9 cases using FNNAC. 6 cases were classified as category III, 5 cases were classified as category IV, and 4 cases were classified as category V. 10 cases underwent surgery for thyroid cancer; lymph node resection was performed at the same time, and the final pathology suggested inflammatory changes. Three cases were diagnosed as tuberculosis through excisional biopsy or CNB, and 1 case was thought to benign for being smaller during about 2 years follow-up. Figure 3 showed a case who was thought to suspicious thyroid cancer metastasis by FNAC but confirmed inflammatory change after surgery.

No statistically significant difference was observed between US-FNAC and US-FNNAC with respect to the age and sex of the four subgroups. Details were shown in Table 2. Table 3 provided the data on the diagnostic performance of the two techniques among the different size groups. The diagnostic accuracy of US-FNAC was superior to that of US-FNNAC in the first three groups (86.4% vs 77.8%, 94.5% vs 85.1%, 98.6% vs 86.0%, respectively), with significant difference in lymph nodes that measured from 5.1 to 10.0 mm (*p* = 0.021) and from 10.1 to 15.0 mm (*p* = 0.011). In lymph nodes >15.0 mm, the accuracy of FNNAC was comparable to that of FNAC, with no significant difference observed between the two techniques. The SEN of FNAC was higher than that of FNNAC in lymph nodes that measured from 5.1 to 10.0 mm (95.8% vs 84.4%) and from 10.1 to 15.0 mm (98.2% vs 83.3%) with statistical significance (*p* = 0.022 and *p* = 0.011). There was no significant difference between the SPE of the two techniques in the four subgroups.

**Table 2.** Baseline characteristics of the enrolled cases.

Subgroups	Age (year) Mean ± SD / median (IQR)	P Value	Sex (Male/Female)	P Value
≤5.0 mm		0.645		0.318
FNAC	45.73 ± 14.59		8/14	
FNNAC	44.21 ± 13.44		21/61	
5.1–10.0 mm		0.590		0.665
FNAC	48.3 ± 15.4		34/74	
FNNAC	45.1 ± 13.4		39/75	
10.1–15.0 mm		0.077		0.783
FNAC	55.9 ± 16.0		29/41	
FNNAC	51.0 ± 14.8		25/32	
>15.0 mm		0.106		0.540
FNAC	58.9 ± 14.4		10/12	
FNNAC	50.4 ± 19.4		8/14	

*FNAC*: fine-needle aspiration cytology; *FNNAC*: fine-needle non-aspiration cytology.

**Table 3.** Diagnostic efficacy of US-FNAC and US-FNNAC in the four subgroups.

Group	FNAC	FNNAC	P Value
Group 1(≤5.0 mm)			
Sensitivity% (95% CI)	84.6(54.6–98.1)	76.9(63.2–87.5)	0.821
Specificity% (95% CI)	88.9 (51.8–99.7)	79.3(60.3–92.0)	0.876
Accuracy% (95% CI)	86.4 (65.1–97.1)	77.8 (67.2–86.3)	0.553
PPV% (95% CI)	91.7 (63.1–98.6)	87.0 (76.3–93.2)	
NPV% (95% CI)	80.0 (52.3–93.6)	65.7 (53.0–76.5)	
Group 2(5.1–10.0 mm)			
Sensitivity% (95% CI)	95.8 (88.1–99.1)	84.4(75.3–91.2)	0.022
Specificity% (95% CI)	92.1 (78.6–98.3)	87.5 (67.6–97.3)	0.669
Accuracy% (95% CI)	94.5(88.4–98.0)	85.1 (77.2–91.1)	0.021
PPV% (95% CI)	95.8 (88.4–98.5)	96.2 (89.8–98.7)	
NPV% (95% CI)	92.1 (79.3–97.3)	60.0(47.5–71.3)	
Group 3(10.1–15.0 mm)			
Sensitivity% (95% CI)	98.2 (90.4–100.0)	83.3(69.8–92.5)	0.011
Specificity% (95% CI)	100.0 (76.8–100.0)	100 (66.4–100.0)	/
Accuracy% (95% CI)	98.6 (92.3–100.0)	86.0(74.2–93.7)	0.011
PPV% (95% CI)	100.0	100.0	
NPV% (95% CI)	93.3 (66.7–99.0)	52.9(37.4–67.9)	
Group 4(>15.0 mm)			
Sensitivity% (95% CI)	95.0(75.1-99.9)	95.2 (76.2–99.9)	1.000
Specificity% (95% CI)	100.0 (15.8–100.0)	100.0 (2.5–100.0)	/
Accuracy% (95% CI)	95.5 (77.2–99.9)	95.5 (77.2–99.9)	/
PPV% (95% CI)	100.0	100.0	
NPV% (95% CI)	66.7 (22.8–93.1)	50.0 (12.9–87.1)	

*FNAC*: fine-needle aspiration cytology; *FNNAC*: fine-needle non-aspiration cytology; *PPV:* positive predictive value; *NPV:* negative predictive value.

Imaging characteristics of the lymphadenopathies were compared between US-FNAC and US-FNNAC in the four subgroups. There were more lymphadenopathies with microcalcification and cystic areas in US-FNNAC than that of US-FNAC (*p* = 0.001 and *p* = 0.002) in lymph nodes from 10.1 to 15.0 mm. Details were shown in Table 4.

**Table 4.** Imaging characteristics of FNAC and FNNAC in the four subgroups.

Imaging characteristics	FNAC	FNNAC	P Value
≤5.0 mm			
Maximum diameter	11.5(4.2)	12.0(4.1)	0.565
Microcalcification	10	20	0.067
Cystic areas			0.871
none	20	74	
≤50%	2	7	
>50%	0	1	
5.1–10.0 mm			
Maximum diameter	16.8 ± 6.2	15.3 ± 5.1	0.053
Microcalcification	36	43	0.495
Cystic areas			0.160
none	94	88	
≤50%	11	21	
>50%	3	5	
10.1–15.0 mm			
Maximum diameter	21.5(6.4)	22.8(6.7)	0.163
Microcalcification	8	24	0.001
Cystic areas			0.002
none	64	38	
≤50%	4	12	
>50%	2	7	
>15.0 mm			
Maximum diameter	30.5(10.3)	30.9(10.6)	1.000
Microcalcification	3	8	0.162
Cystic areas			0.310
none	17	12	
≤50%	4	5	
>50%	1	5	

*FNAC*: fine-needle aspiration cytology; *FNNAC*: fine-needle non-aspiration cytology.

No significant complications such as bleeding and tumor seeding occurred during the US-FNC procedure. 5 cases complicated pain but disappeared at follow-up evaluation.

## 4. Discussion

FNAC is a widely acknowledged approach for assessing thyroid nodules and lymphadenopathies. However, this method often encounters the issue of blood-contaminated samples, which can lead to challenges in interpreting the cytologic findings. In contrast to FNAC, which employs relatively high suction pressures during sample collection, FNNAC is a non-aspiration technique that relies on capillary force within narrow channels. It has been proposed that FNNAC may yield superior quality samples by reducing cell dilution caused by blood [17]. However, FNNAC may not yield adequate specimens as FNAC and there was no agreement on which technique had better diagnostic performance. The decision to choose either FNAC or FNNAC was mainly based on individual experience. This research aimed to compare the diagnostic value of US-FNAC and US-FNNAC for lymphadenopathies in the neck with a relatively large sample size and with emphasis on the influence of lesion size.

The proportion of specimens with non-diagnostic results (categorized as Sydney System Category I) was 0.6%, demonstrating a lower occurrence compared to the rates of inadequately sampled specimens documented in earlier research [5,20,21]. This may be due to the ultrasound guidance which can help the operator avoid the cystic areas or calcification. In addition, the specimens of most enrolled cases undertaken smear cytology, liquid-based cytology and cell block. The above three cytological diagnostic methods complemented each other and greatly improved the diagnostic efficiency.

Regarding the overall diagnostic performance of US-FNC based on the Sydney System, we observed SEN of 88.7%, SPE of 89.7%, PPV of 96.2%, NPV of 72.9%, and accuracy of 88.9%, which were lower than the existed studies, especially for SPE [5,20,21]. In most published studies, the cytologic diagnoses of category I and category III were removed when calculating the overall diagnostic performance. Thus, we reanalysed the diagnostic value of US-FNC only in begin, suspicious and malignant cases, the SEN, SPE, PPV, NPV and accuracy of the Sydney System turned to 88.5%, 93.3%, 97.6%, 72.7% and 89.7%, with a significant improvement in SPE. In the 9 false-positive cases which were classified as category IV or category V, 8 cases were misdiagnosed as metastasis of thyroid cancer but thought to inflammatory after surgery, of which 6 lymph nodes with the smallest dimension ≤ 5.0 mm. These tiny lymph nodes pose a challenge for aspiration and sampling *via* FNC, complicating the interpretation of cytology results [22]. Thyroglobulin in FNC may improve the diagnostic efficacy in these cases [23]. The other 1 case was misdiagnosed as tumour with unknown resources, but CNB showed tuberculosis finally. Studies have shown that FNC has a lower SPE and significantly lower precision than GeneXpert in correctly identifying cases with extrapulmonary tuberculosis because it exhibits similar cytomorphological characteristics with lesions that are not associated with tuberculosis, so GeneXpert and Ziehl-Neelsen stain should be utilized with FNC to diagnose those suspected extrapulmonary tuberculosis [24,25]. Due to the need for sufficient samples to conduct the aforementioned supplementary tests, FNAC has been predominantly utilized in existing studies [23,24]. Meanwhile, our study demonstrated no significant variance in diagnostic efficacy between FNAC and FNNAC in lymph nodes ≤5.0 mm, which indicating that FNAC is more suitable for assessing these lymphadenopathies.

FNAC demonstrated higher overall diagnostic SEN and accuracy compared to FNNAC, although no statistically significant difference was observed in SPE. In the size subgroups, our data revealed that US-FNAC acquired significant better diagnostic performance than US-FNNAC in lymph nodes that measured from 5.1 to 10.0 mm and that measured from 10.1 to 15.0 mm. No statistically significant difference was observed between the diagnostic efficacy of the two techniques in the other two groups. The above results indicated that US-FNAC could be applied preferentially to that US-FNNAC sampling for cervical lymphadenopathies, especially for those lesions that measured from 5.1 mm to 10.0 mm and 10.1 to 15.0 mm. Such conclusion was somewhat in concordance with the study of Zhou et al, who observed that US-FNNAC was able to provide better sample material in thyroid nodules that measured from 10.1 to 20.0 mm than in nodules that measured ≤5.0 mm or from 5.1 to 10.0 mm with respect to scores for the number of obtained cells, preserved tissue architecture, and cellular degeneration [15]. As a tertiary hospital, our study included numerous cases of lymphatic tuberculosis, lymphoma, lung cancer and breast cancer metastases. These cases necessitated more extensive tissue sampling for ICC to elucidate the nature and origin of the lesions. For larger lymph nodes (those exceeding 15.0 mm), both FNAC and FNNAC can effectively sample a broader representative area of tissue, thereby increasing the likelihood of obtaining adequate samples for such nodes [26]. In our study, the two techniques showed good diagnostic performance in these lymphadenopathies. While for lymph nodes measured from 5.1 to 10.0 mm and 10.1 to 15.0 mm, FNAC with suction may have a potential benefit in obtaining sufficient samples for accurate differential diagnosis of benign and malignant lesions. However, our results were somewhat in discordance with the previous study of Xia et al[17]. The lymph nodes in their study were divided into 2 size groups based on the smallest dimension: <6.0 mm and ≥6.0 mm and the results indicated that no significant difference were seen between FNAC and FNNAC in the two size subgroups [17]. Variations in the study subjects and sample sizes may have contributed to incongruent findings, in which they focused on the evaluation of lymph node metastasis of thyroid cancer with a relatively small sample size while we focused on all the cervical lymphadenopathies with a large sample size, thus further prospective large-scale studies are necessary to validate our results.

We also compared the ultrasonic characteristics between US-FNAC and US-FNNAC in size subgroups. In lymphadenopathies that measured from 10.1 mm to 15.0 mm, there were more cases with microcalcification and cystic areas in FNNAC than that of FNAC (*p* = 0.001 and *p* = 0.002). While microcalcifications in lymph nodes are highly specific for metastatic papillary thyroid carcinoma, their presence may obscure cellularity in cytological samples. Similarly, cystic areas increase non-diagnostic rates due to acellular fluid or degenerated cells, as reported in thyroid nodules by Cengic et al, who demonstrated that predominantly cystic lesions require more passes to achieve specimen adequacy [27]. However, during US-guided procedures, operators actively avoided cystic and calcified areas to prioritize sampling of solid components, thereby mitigating potential confounding effects.

There were several limitations in this study. First, as a retrospective study conducted at a single center, there may be potential selection bias. Second, although the total sample size included up to 500 lymphadenopathies, some size-based subgroups, particularly lymph nodes measuring >15.0 mm, had relatively small sample sizes. This is likely because many patients with such lesions underwent US-CNB at our center, with only those located near major cervical vessels being evaluated using US-FNC. In addition, we did not assess inter-operator variability among the radiologists who performed the sampling procedures, which could influence the consistency of the results.

## Conclusions

In summary, our results supported the preferential use of US-FNAC over US-FNNAC in routine clinical practice for lymph node evaluation, particularly in lymph nodes measuring 5.1–15.0 mm. Additional tests such as thyroglobulin were necessary to improve the diagnostic performance of US-FNC in lymph nodes that measured ≤5.0 mm.

## Data Availability

The datasets used and/or analysed during the current study are available from the corresponding author on reasonable request.

## References

[1] Caraway NP. Strategies to diagnose lymphoproliferative disorders by fine-needle aspiration by using ancillary studies. Cancer. 2005;105(6):432–442. doi: 10.1002/cncr.21452. 16222688

[2] Angelico G, Santoro A, Inzani F, et al. Ultrasound-guided FNA cytology of groin lymph nodes improves the management of squamous cell carcinoma of the vulva: results from a comparative cytohistological study. Cancer Cytopathol. 2019;127(8):514–520. doi: 10.1002/cncy.22154. 31174235

[3] Lachar WA, Shahab I, Saad AJ. Accuracy and cost-effectiveness of core needle biopsy in the evaluation of suspected lymphoma: a study of 101 cases. Arch Pathol Lab Med. 2007;131(7):1033–1039. doi: 10.5858/2007-131-1033-AACOCN. 17616988

[4] Han F, Xu M, Xie T, et al. Efficacy of ultrasound-guided core needle biopsy in cervical lymphadenopathy: a retrospective study of 6,695 cases. Eur Radiol. 2018;28(5):1809–1817. doi: 10.1007/s00330-017-5116-1. 29188372

[5] Fu Y, Liu C, Ren M, et al. Accuracy of ultrasound-guided fine-needle aspiration for small cervical lymph nodes: a retrospective review of 505 cases. Heliyon. 2024;10(10):e31238. doi: 10.1016/j.heliyon.2024.e31238. 38803905 PMC11128987

[6] Vigliar E, Acanfora G, Iaccarino A, et al. A novel approach to classification and reporting of lymph node fine-needle cytology: application of the proposed Sydney system. Diagnostics (Basel). 2021;11(8) :1314. doi: 10.3390/diagnostics11081314. PMC8393909 34441249

[7] Gupta P, Gupta N, Kumar P, et al. Assessment of risk of malignancy by application of the proposed sydney system for classification and reporting lymph node cytopathology. Cancer Cytopathol. 2021;129(9):701–718. doi: 10.1002/cncy.22432. 33830657

[8] Romitelli F, Di Stasio E, Santoro C, et al. A comparative study of fine needle aspiration and fine needle non-aspiration biopsy on suspected thyroid nodules. Endocr Pathol. 2009;Summer20(2):108–113. doi: 10.1007/s12022-009-9074-2. 19377844

[9] Kamal MM, Arjune DG, Kulkarni HR. Comparative study of fine needle aspiration and fine needle capillary sampling of thyroid lesions. Acta Cytol. 2002;46(1):30–34. doi: 10.1159/000326712. 11843555

[10] Briffod M, Gentile A, Hébert H. Cytopuncture in the follow-up of breast carcinoma. Acta Cytol. 1982;26(2):195–200. 6952722

[11] Srikanth S, Anandam G, Kashif MM. A comparative study of fine-needle aspiration and fine-needle non-aspiration techniques in head and neck swellings. Indian J Cancer. 2014;51(2):98–99. doi: 10.4103/0019-509X.137935. 25104186

[12] Mair S, Dunbar F, Becker PJ, et al. Du Plessis W. Fine needle cytology–is aspiration suction necessary? A study of 100 masses in various sites. Acta Cytol. 1989;33(6):809–813. 2488680

[13] Hatami H, Samsami M, Movahedinia S, et al. Comparison of fine-needle aspiration with fine-needle capillary cytology in thyroid nodules. Ann R Coll Surg Engl. 2023;105(2):162–165. doi: 10.1308/rcsann.2021.0367. 35446712 PMC9889172

[14] Wang D, Fu HJ, Xu HX, et al. Comparison of fine needle aspiration and non-aspiration cytology for diagnosis of thyroid nodules: a prospective, randomized, and controlled trial. Clin Hemorheol Microcirc. 2017;66(1):67–81. doi: 10.3233/CH-160222. 28128748

[15] Zhou JQ, Zhang JW, Zhan WW, et al. Comparison of fine-needle aspiration and fine-needle capillary sampling of thyroid nodules: a prospective study with emphasis on the influence of nodule size. Cancer Cytopathol. 2014;122(4):266–273. doi: 10.1002/cncy.21382. 24302655

[16] Pinki P, Alok D, Ranjan A, et al. Fine needle aspiration cytology versus fine needle capillary sampling in cytological diagnosis of thyroid lesions. Iran J Pathol. 2015;Winter10(1):47–53. 26516325 PMC4539790

[17] Xia S, Chen Y, Zhan W, et al. Ultrasound-guided fine-needle aspiration versus fine-needle capillary sampling in evaluation of lymph node metastasis of thyroid cancer. Front Oncol. 2021;11:642142. doi: 10.3389/fonc.2021.642142. 33937044 PMC8079778

[18] Whitlock J, Taeymans O, Monti P. A comparison of cytological quality between fine-needle aspiration and non-aspiration techniques for obtaining ultrasound-guided samples from canine and feline lymph nodes. Vet Rec. 2021;188(6):e25. doi: 10.1002/vetr.25. 33729570

[19] Al-Abbadi MA, Barroca H, Bode-Lesniewska B, et al. A proposal for the performance, classification, and reporting of lymph node fine-needle aspiration cytopathology: the Sydney System. Acta Cytol. 2020;64(4):306–322. doi: 10.1159/000506497. 32454496

[20] Bueno AP, Palu RF, Dalcin JF, et al. Accuracy of fine-needle aspiration of lymph nodes: a cancer center’s experience. Cytopathology. 2022;33(1):114–118. doi: 10.1111/cyt.13057. 34528327

[21] Houcine Y, Romdhane E, Blel A, et al. Evaluation of fine needle aspiration cytology in the diagnosis of cervical lymph node lymphomas. J Craniomaxillofac Surg. 2018;46(7):1117–1120. doi: 10.1016/j.jcms.2018.04.024. 29779620

[22] Hall TL, Layfield LJ, Philippe A, et al. Sources of diagnostic error in fine needle aspiration of the thyroid. Cancer. 1989;63(4):718–725. doi: 10.1002/1097-0142(19890215)63:4<718::AID-CNCR2820630420>3.0.CO;2-N. 2914278

[23] Wang Y, Duan Y, Li H, et al. Detection of thyroglobulin in fine-needle aspiration for diagnosis of metastatic lateral cervical lymph nodes in papillary thyroid carcinoma: a retrospective study. Front Oncol. 2022;12:909723. doi: 10.3389/fonc.2022.909723. 36203449 PMC9530248

[24] Dharwadkar A, Ingale Y, Deokar N, et al. Significance of various diagnostic modalities in detection of tuberculosis in cervical lymphadenopathy: a study of 200 cases. Int J Mycobacteriol. 2024;13(2):171–177. doi: 10.4103/ijmy.ijmy_45_24. 38916388

[25] Kumbi H, Ali MM, Abate A. Performance of fine needle aspiration cytology and ziehl-neelsen staining technique in the diagnosis of tuberculosis lymphadenitis. BMC Infect Dis. 2024;24(1):633. doi: 10.1186/s12879-024-09554-z. 38918686 PMC11197254

[26] He Y, Ji X, Xie Y, et al. Clinical application of ultrasound-guided core needle biopsy with multiple punches in the diagnosis of lymphoma. World J Surg Oncol. 2015;13(1):126. doi: 10.1186/s12957-015-0537-2. 25885784 PMC4383197

[27] Cengic I, Tureli D, Altas H, et al. Effects of nodule characteristics on sampling number and duration of thyroid fine-needle aspiration biopsy: size does not matter, but cystic degeneration ratio does. Acta Radiol. 2017;58(3):286–291. doi: 10.1177/0284185116649797. 27235454

